# Transcriptomes of cochlear inner and outer hair cells from adult mice

**DOI:** 10.1038/sdata.2018.199

**Published:** 2018-10-02

**Authors:** Yi Li, Huizhan Liu, Kimberlee P. Giffen, Lei Chen, Kirk W. Beisel, David Z. Z. He

**Affiliations:** 1 Department of Otorhinolaryngology, Beijing Tongren Hospital, Beijing Capital Medical University, Beijing 100730, China; 2 Department of Biomedical Sciences, Creighton University School of Medicine, Omaha, Nebraska 68170, USA; 3 Chongqing Academy of Animal Science, Chongqing 402460, China

**Keywords:** RNA sequencing, Gene expression, Hair cell

## Abstract

Inner hair cells (IHCs) and outer hair cells (OHCs) are the two anatomically and functionally distinct types of mechanosensitive receptor cells in the mammalian cochlea. The molecular mechanisms defining their morphological and functional specializations are largely unclear. As a first step to uncover the underlying mechanisms, we examined the transcriptomes of IHCs and OHCs isolated from adult CBA/J mouse cochleae. One thousand IHCs and OHCs were separately collected using the suction pipette technique. RNA sequencing of IHCs and OHCs was performed and their transcriptomes were analyzed. The results were validated by comparing some IHC and OHC preferentially expressed genes between present study and published microarray-based data as well as by real-time qPCR. Antibody-based immunocytochemistry was used to validate preferential expression of SLC7A14 and DNM3 in IHCs and OHCs. These data are expected to serve as a highly valuable resource for unraveling the molecular mechanisms underlying different biological properties of IHCs and OHCs as well as to provide a road map for future characterization of genes expressed in IHCs and OHCs.

## Background & Summary

Hair cells are the sensory receptors of both the auditory system and the vestibular system in the ears of all vertebrates. Hair cells transduce mechanical stimuli, i.e., movement in their environment, into electrical activity^1,2^. There are two types of hair cells in the mammalian cochlea, inner hair cells (IHCs) and outer hair cells (OHCs). These two types of hair cells are anatomically and functionally distinct^3^. Although much is known about how IHCs and OHCs function in hearing, we have limited knowledge of molecular mechanisms, i.e., gene expression and regulation, that underlie their distinct morphological and functional specializations.

While all cells in multicellular organisms have nearly identical genome, the genes that are transcribed are different for each cell type. Diverse patterns of gene expression and post-transcriptional regulation of gene expression by miRNA underlie phenotypic variances of different cell types. Genome-wide characterization of cell-specific transcriptomes is central to understanding the biological property of a cell or a population of cells. High-throughput mRNA sequencing (RNA-seq) allows simultaneous transcript discovery and abundance estimation with a wide dynamic range and lower false-negative and false-positive discovery rates^4,5^. Direct sequencing of RNA libraries also provides the opportunity to explore alternative splicing, a key mechanism that contributes to transcriptome diversity^6–8^. Transcriptome analysis has emerged as a powerful tool in revealing the genetic and molecular profile of a cell or a population of cells.

In a previous study, we used the microarray technique to examine transcriptional profiling of purified IHCs and OHCs from adult mice^9^. Although microarray is a powerful technique, it has limitations in dynamic range and identification of multiple splice variants of the genes. Furthermore, it relies on prerequisite sequence information, which precludes analysis of unannotated genes^10^. Because of this, 22 to 24% of the transcripts detected in our previous microarray study were uncharacterized or unannotated transcripts or genes^9^. Hair cell-specific transcriptomes have been analyzed using RNA-seq in several recent studies^11–14^. However, these studies analyzed transcriptomes of cochlear and vestibular hair cells only from embryonic and neonatal mice. Furthermore, those studies did not distinguish between IHCs and OHCs.

**Figure 1:**
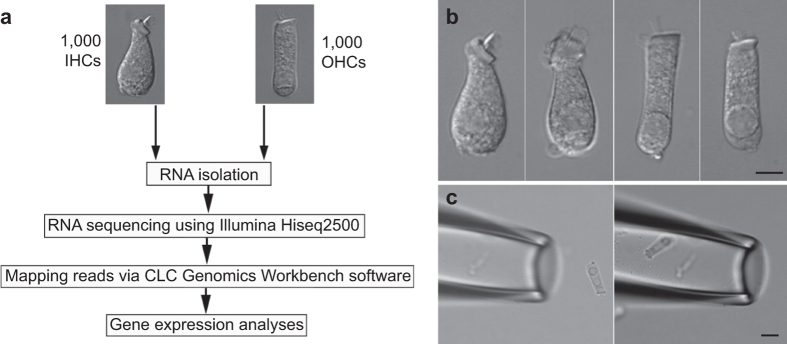
Study design workflow for RNA-seq and suction pipette technique for collecting isolated hair cells. (**a**) Workflow of experimental design for RNA-seq transcriptomic analysis of IHCs and OHCs isolated from adult mouse cochleae. (**b**) Representative images (from left to right) of two isolated IHCs and two OHCs from adult mice. (**c**) A pick-up pipette before and after an isolated OHC was drawn into the pipette. This technique was used to individually collect isolated hair cells. Bars: 5 (**b**) and 10 (**c**) μm.

Here, we describe transcriptome-wide profiling of IHCs and OHCs obtained from one-month-old CBA/J mouse cochleae to provide a comprehensive view of the gene expression in IHCs and OHCs. Unlike some other mouse strains (such as C57/B6) which carry mutations that can cause early onset of age-related hearing loss, CBA/J mice do not exhibit age-related hearing loss until 18 months of age. We took advantage of the established pulled glass pipette technique^9,15^ and distinct morphology of the two types of hair cells to separately collect 1,000 isolated IHCs and OHCs. Two biological replicates of IHCs and three replicates of OHCs, each containing 1,000 hair cells, were prepared for RNA-seq. An overview of the study design is depicted in Fig. 1a. Transcriptomes of adult IHCs and OHCs from microarray technique^9^, as well as neonatal hair cells from RNA-seq^11–14^ were presented along with transcriptomes from the current study. We validated our results by comparing some IHC and OHC preferentially expressed genes between the present study and previous studies^9,11–14^ as well as by real-time quantitative PCR (RT qPCR). In addition, we used antibody-based immunostaining to show the preferential expression of SLC7A14 and DNM3, whose function in hair cells has not been characterized. While SLC7A14 showed strong staining in the soma of IHCs, DNM3 was detected in the stereocilia bundle of only OHCs. These two genes/proteins can be used as specific markers for adult IHCs and OHCs. Finally, we examined the expression of deafness-related genes in hair cells. Mutations or deficiencies affecting approximately 150 genes have been linked to inherited syndromic or non-syndromic hearing loss^16^. We analyzed the expression of 143 known deafness genes, excluding X-chromosome-linked genes, and showed 128 genes are expressed in hair cells.

Our dataset is expected to serve as a highly valuable resource for unraveling the molecular mechanisms underlying different biological properties of IHCs and OHCs. The dataset will also provide a road map for future characterization of genes expressed in these two types of hair cells and for assisting the auditory research community in exploring the functions of deafness-related genes.

## Methods

### Hair cell isolation and collection

CBA/J mice aged between 28 and 35 days old were used for the study. The basilar membrane together with the organ of Corti was isolated as described before^15^. The sensory epithelium was transferred to an enzymatic digestion medium containing 1 ml L-15 and 1 mg Collagenase IV (Sigma) in a small Petri dish. After 5 min for incubation at room temperature (20 ± 2 ^o^C), the tissue was transferred to a small plastic chamber (0.8 ml in volume) containing enzyme-free Leibovitz’s L-15 medium (7.35 pH, 300 mOsm). Hair cells were separated after gentle trituration of the basilar membrane with a 200 μL Eppendorf pipette tip. The chamber containing the hair cells was then mounted onto the stage of an inverted Olympus IX71 microscope equipped with a video camera. The chamber (with inlet and outlet) was perfused with fresh L-15 medium to wash out debris for 5 min. IHCs and OHCs in most cases retained their distinct morphological feature after isolation. Some representative images of solitary IHCs and OHCs are presented in Fig. 1b.

To collect solitary hair cells, two pulled glass pipettes with a diameter of ~30 μm were used to pick up and transfer IHCs and OHCs. Each pipette was designated for one cell type to prevent cell type contamination in the pipette. The pickup pipette was fabricated from 1.5 mm thin-wall glass tubing pulled by a two-stage electrode puller. The pipettes were mounted in two separate electrode holders mounted on two Narashigi micromanipulators (Narashigi, Japan). The suction port of the pipette holder, held by the micromanipulator, was connected to a micrometer-driven syringe to provide positive or negative pressure to draw in or expel the cells. An image of an OHC before being drawn into a pickup pipette is shown in Fig. 1c. A video showing a mouse OHC being drawing into a pickup pipette is provided (Data Citation 1). IHCs and OHCs were identified based on their morphology under direct visual observation and solitary hair cells that were not attached to any other cell types were collected. Any hair cells with ambiguous morphology were excluded. Hair cells were transferred to a microcentrifuge tube containing 50 μl RNA*later* (Thermo Fisher Scientific, Waltham, MA) after ~10 cells were collected in the pipette. Cells were expelled from the pipette by applying positive pressure. This step was repeated until approximately 50 to 80 IHCs and 100 to 150 OHCs were collected from each mouse. Thirty mice were used for the collection of two biological replicates of IHCs and three replicates of OHCs.

### RNA isolation, amplification

Approximately 1,000 cells suspended in 100 μL RNA*Later* from each biological replicate were used to extract total RNA, including small RNAs (>~18 nucleotides), using the Qiagen miRNeasy mini plus Kit (Qiagen Sciences Inc, Germantown, MD). DNA contamination was eliminated by on-column DNase digestion. The quality and quantity of RNA after purification was examined using an Agilent 2100 BioAnalyzer (Agilent Technologies, Santa Clara, CA) and compared to examples of pure RNA results found in the Agilent 2100 Bioanalyzer 2100 Expert User’s Guide. Total RNA from each sample was approximately 8 to 10 ng/μl (with ~3–4 μl total for each sample). These samples were reverse transcribed into cDNA and amplified using the SMART-Seq V4 Ultra Low Input RNA kit (Clontech Laboratories, Inc., Mountain View, CA).

### RNA-sequencing and bioinformatic analyses

Genome-wide transcriptome libraries were produced from biological replicates of IHCs and OHCs. SMART-Seq V4 Ultra Low Input RNA kit (Clontech) was used to generate cDNA in combination with the Nextera Library preparation kit (Illumina, Inc., San Diego, CA). To ensure the inserts were the appropriate size and to determine concentration prior to sequencing, a Bioanalyzer 2100 and a Qubit fluorometer (Invitrogen) were used to assess library size and concentration. Transcriptome libraries were sequenced using the HiSeq 2500 Sequencing System (Illumina). Libraries were multiplexed and three samples per lane were sequenced as 100-bp paired-end reads. This generated approximately 100 million reads per sample. The files from the multiplexed RNA-seq samples were demulitplexed and fastq files representing each library and quality control data were generated.

### Bioinformatics analyses

CLC Genomics Workbench software (CLC bio, Waltham, MA, USA) was used to map the reads to the mouse genome (mm10, build name GRCm38) and generate gene expression values in the normalized form of reads per kilobase of transcript per million mapped reads (RPKM) values. Reads were mapped to exonic, intronic, and intergenic sections of the genome. Gene expression estimates were derived from the mapped reads using HTSeq count^17^. Ingenuity IPA program (www.ingenuity.com) and DAVID^18^ were used for functional annotation. Entrez Gene, HGNC, OMIM, and Ensembl database were used for verification, reference, and analyses.

### Code availability

No custom code was used in any of these analyses.

### Real-time qPCR

We validated the expression of 26 genes using RT qPCR. RT qPCR experiments were run on an Applied Biosystems 7500 Fast Real-Time PCR system. Ten microliters of Powerup SYBR Green Master Mix (Thermo Fisher Scientific, Waltham, MA, USA) was used in each 20 microliter reaction. Primer concentrations were 450 nM. The original cDNA samples were diluted twenty-fold with two microliters for every reaction. The fast thermal cycling mode of the Applied Biosystems 7500 instrument was used. We calculated ∆Ct values (∆Ct = Ct^(GOI)^ − Ct^AVG HKG^) of each gene (gene of interest or GOI) after normalizing to Ct value of a house-keeping gene (HKG). For comparing differential expression of a gene between IHCs and OHCs, we calculated ∆∆Ct, where ∆∆Ct = ∆Ct (IHCs) − ∆Ct (OHCs)^19^. Thus, a positive value would suggest that this gene has a higher expression value in IHCs than OHCs, whereas a negative value suggesting higher expression in OHCs than in IHCs.

The sequences of the oligonucleotide primers were designed using A plasmid Editor (ApE) software (http://biologylabs.utah.edu/jorgensen/wayned/ape/) and BLAST searches (http://blast.ncbinlm.nih.gov/Blast.cgi.) to find unique and appropriate sequences with melting temperatures above 60 °C that had predicted low rates of homodimerization. Oligonucleotide primers were acquired from Integrated DNA Technologies (Coralville, Iowa). The sequences of oligonucleotide primers are shown in Table 1.

### Immunocytochemistry

Cochleae were perfused with 4% formaldehyde in phosphate buffered saline (PBS) and the basilar membrane together with the organ of Corti was dissected out. The tissue was treated with 0.2% Triton X-100/PBS and goat serum (10%) was used to block nonspecific binding. The tissue was then incubated with antibodies (anti-Slc7a14: HPA045929, Lot: R43519 from Atlas Antibodies; anti-Dnm3: AB3458, Lot: GR154711-3 from Abcam) and washed with PBS, followed by incubation with secondary antibodies (Life Technologies, Lot# 1579044). The samples were mounted on glass slides with antifade solution (Prolong Antifade Kit, Invitrogen, Carlsbad, CA) before imaging on a Leica Confocal Microscope (Leica TCS SP8 MP). Three cochleae from three adult CBA/J mice were used for immunodetection.

## Data Records

Raw fastq sequencing files, comprised of 2 biological repeats of IHCs and 3 biological repeats of OHCs, each with 2 technical repeats, have been deposited in the NCBI Sequence Read Archive (Data Citation 2). The individual accession numbers for each biological and technical replicate is provided in Table 2. An excel file containing the RPKM gene expression values of each biological and technical repeat of IHCs and OHCs is included as “GSE111348_Inner_and_outer_ hair_cells_RPKM.xlsx” (Data Citation 3). Since microarray-based transcriptomes of IHCs and OHCs from adult CBA/J mice are available from our previous study (Data Citation 4)^9^, we aligned the expression values of all the genes detected from RNA-seq and microarray, according to the Ensembl annotated gene names (symbols). We also obtained transcriptome datasets (Data Citation 5 and Data Citation 6) of neonatal cochlear hair cells from two published studies^12,13^. The gene expression values together with transcriptome datasets from these published studies are included for comparison in Data Citation 7. Alignment of each gene from different studies was also assisted by reference to Ensembl, HGNC, Entrez Gene and OMIM. Additional resources such as the gEAR (https://www.umgear.org) and SHIELD (https://shield.hms.harvard.edu/index.html) were also used for reference and verification.

## Technical Validation

### RNA quality control and RNA-seq quality control

We analyzed RNA quality and concentration of our samples to determine their suitability for RNA-sequencing using an Agilent 2100 BioAnalyzer. In addition to using the 2:1 ratio (28S:18S) as an indication for determining the integrity of RNA in the electropherogram, we also used the RIN (RNA integrity number) software algorithm to evaluate the quality of our RNA samples. All of our samples had a RIN of 9, indicating that the integrity of RNA samples was high with minimal degradation.

### Sequencing accuracy

We used the FastQC app (version 1.0.0) on the Illumina cloud computing interface (https://basespace.illumina.com/ome/index) to examine the quality of the reads. The analysis compared the read signals to the probability of accurate base-reading with a Phred quality score^20^. The fastq files generated from RNA-sequencing were analyzed for base-reading accuracy. All our sequencing runs exceeded 30, which reflects a 99.9% accuracy of the correct base at a given nucleotide in the sequence. This suggests that the RNA-sequencing performed was of high quality and unambiguous. We used Phred quality score ≥ 30 as the high-quality cutoff in our analysis for all samples.

### Reproducibility of biological samples

**Figure 2:**
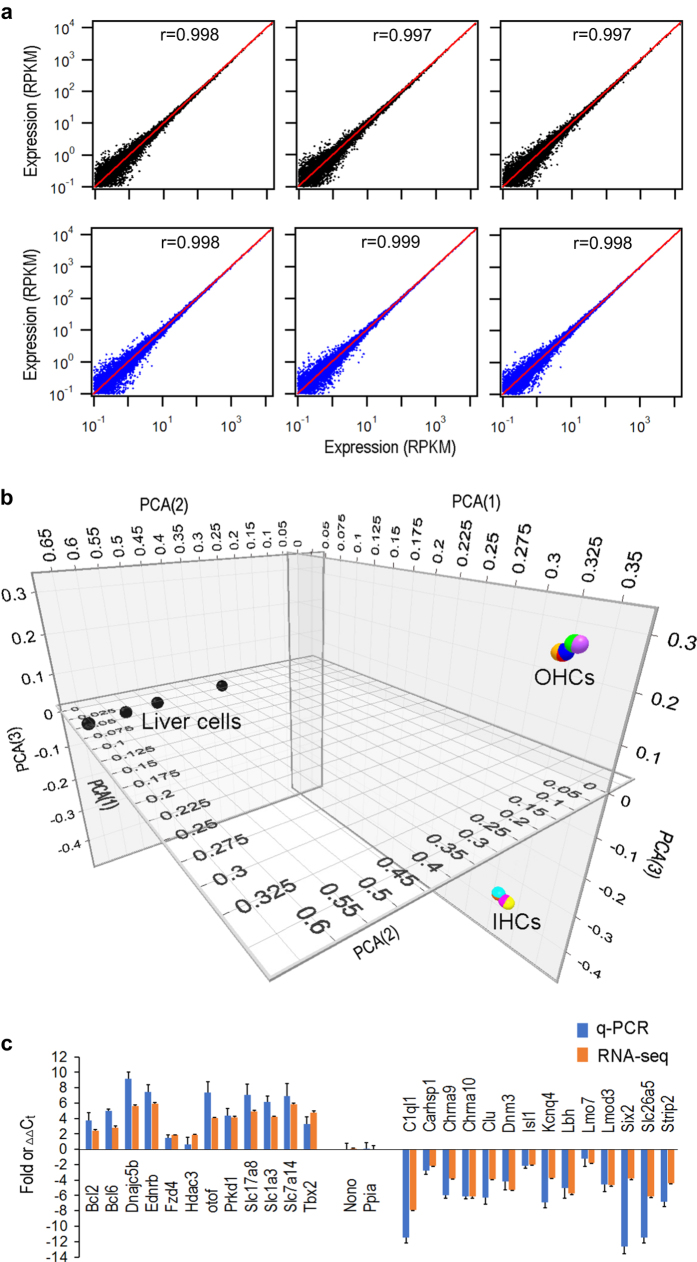
Reproducibility of biological replicates and RT qPCR validation of differential expression of 27 genes in IHCs and OHCs. (**a**) Correlation coefficient between biological replicates from OHCs. Correlation coefficient (r) for each comparison is given in each panel. (**b**) Correlation coefficient between technical repeats of RNA-seq from OHC samples. (**c**) PCA analysis of the gene expression profiles of IHCs and OHCs compared with liver cells. Filled circles with different colors represent different biological and technical repeats for IHCs and OHCs. (**d**) Validation of differential expression of 26 genes between IHCs and OHCs using RT qPCR and RNA-seq. Positive values indicate higher expression of the genes in IHCs than in OHCs, while negative values indicate higher expression the genes in OHCs than in IHCs. Fold differences, based from RPKM values from RNA-seq, are all calculated in log2 base. ∆∆Ct for each gene was calculated from RT qPCR.

Correlation coefficient was used to examine reproducibility of biological and technical replicates of IHCs and OHCs. Fig. 2a presents three plots of comparison among three biological replicates OHCs while Fig. 2b shows the comparison among three technical repeats of the dataset for OHCs. As shown, the data points are all concentrated near the line (replicate 1) with small deviation. The mean correlation coefficient between biological replicates of OHCs is 0.994 ± 0.0003 (mean ± SD), while the mean correlation coefficient between technical repeats of OHCs is 0.999 ± 0.0045. The correlation coefficient between biological replicates of IHCs is 0.9984 ± 0.0003 (mean ± SD), and the coefficient between technical repeats is 0.994 ± 0.0045. The analysis suggests that the results were highly reproducible.

**Figure 3:**
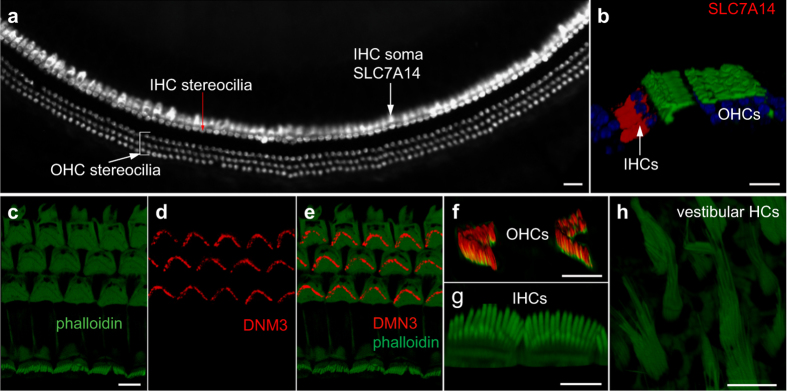
Differential expression of SLC7A14 and DNM3 in IHCs and OHCs by immunocytochemistry and confocal microscopy. (**a**) Expression of SLC7A14 in IHCs in a whole mount preparation from an adult mouse cochlea. Stereocilia bundles were labelled with rhodamine-phalloidin while SLC7A14 was labelled with anti-SLC7A14 antibody. Bar: 20 μm. (**b**) Confocal optical sectioning from the whole mount preparation in (**a**). Expression of SLC7A14 (in red) is only detected in IHCs. Bar: 10 μm. (**c**) Top view of stereocilia bundles of hair cells. Rhodamine-phalloidin (in green) was used to label actin. Bar: 5 μm (for c, d and e). (**d**) Expression of DNM3 (in red) in the same view as in **c**. (**e**) A merged image of c and d. (**f**): Optical sectioning image of OHC stereocilia bundles under high magnification. (**g**) Optical sectioning image of IHC stereocilia bundles. (**h**) Image of vestibular hair cells. In f, g and h, antibodies against phalloidin and DNM3 were used. Expression of DNM3 was only seen in the stereocilia bundle of OHCs. Bar: 5 μm for f and g and h.

Principal component analysis (PCA) is a technique commonly used to measure levels of variation and similarity among gene expression datasets. We used PCA to examine similarity of gene expression of different cell populations as well as reproducibility of biological replicates. Fig. 3c shows PCA of the gene expression profiles of IHCs and OHCs. Transcriptome data of mouse liver cells from a published study^21^ was downloaded and normalized with our data set. As shown, the expression profiles of OHCs are highly reproducible as the data points from three biological and three technical repeats are clustered all together with small variability. Similarly, the expression profiles of IHCs are also highly reproducible. However, the datasets of IHCs and OHCs are separated by a large distance, suggesting that their gene expression profiles are different. The gene expression profile of liver cells is also distinct from those of IHCs and OHCs, as liver cells are further away from hair cells in the graph.

### Real-time qPCR validation

Fifteen additional CBA/J mice were used to prepare three biological replicates of IHCs and OHCs for RT qPCR to validate the expression of 26 genes, 14 of which were highly expressed in OHCs and 12 were highly expressed in IHCs. The expression values were all normalized to the cycle threshold (Ct) value of *Nono* and *Ppia*. *Nono* and *Ppia*, used as reference genes in a previous study^19^, had similar level of expression with no statistical significance between the two populations of hair cells in both previous microarray^9^ and present RNA-seq studies. We compared the patterns of differential expression of these genes between IHCs and OHCs using expression values from qPCR and RNA-seq. While log2 fold difference for each gene was computed using the RPKM values of IHCs vs. OHCs from RNA-seq, the ∆∆Ct for each gene was calculated from RT qPCR. Fig. 2d shows such a comparison after the expression values were normalized to fold changes. Although the values from two analyses are different, the trend of differential expression of these genes is highly consistent between the two datasets.

### Immunocytochemistry

We used immunocytochemistry to detect the expression of SLC7A14 and DNM3; the function of these proteins in the two populations of hair cells has not been characterized. *Slc7a14* and *Dnm3* are differentially expressed in IHCs and OHCs, respectively, as shown in our previous microarray-based transcriptome analysis^9^. Current study (Fig. 2d) also shows that *Slc7a14* and *Dnm3* are preferentially expressed in IHCs and OHCs, respectively. *Slc7a14* is predicted to encode a glycosylated, cationic amino acid transporter protein to mediate lysosomal uptake of cationic amino acids. This gene is expressed in the photoreceptor layer of the retina and mutations in this gene are associated with autosomal recessive retinitis pigmentosa^22^. *Dnm3* encodes dynamin-3, which is predicated to be involved in producing microtubule bundles and able to bind and hydrolyze GTP. We used antibodies (against SLC7A14 and DNM3) and confocal microscopy to determine where they are expressed and whether they are differentially expressed. As shown in Fig. 3a,b, strong staining of SLC7A14 is detected in the soma of IHCs and but not in the soma of OHCs. Thus, SLC7A14 may be used as a specific marker for IHCs. Conversely, DNM3 expression is detected in the stereocilia bundle of OHCs, but not in the bundle of IHCs and vestibular hair cells (Fig. 3c–h) suggesting that DNM3 may play an important role in the biological property of OHC stereocilia and the components of the IHC and OHC stereocilia may be different. The functional roles of these two proteins in OHCs and IHCs are yet to be determined.

### Validation by comparison with published studies

Previous studies have identified and characterized many genes expressed in hair cells in developing and adult animals using immunocytochemistry, molecular biology, and electrophysiology techniques. These genes encode some proteins for unique structure and function of hair cells as well as transcription factors important for hair cell differentiation, specification and maintenance. Since the expression of these genes has been validated by either *in situ* hybridization, antibody staining or molecular biology and electrophysiology techniques, comparison of the genes detected in our RNA-seq analysis with the genes that are already be described in the inner ear is a good way to validate our dataset. We compiled a list of genes that were identified in previous studies and presented in Table 3 (available online only). In the table, the expression (RPKM) values from our RNA-seq analysis are included for comparison. As shown, most genes that were previously detected in hair cells are also expressed in our dataset. We should point out though, some genes (especially those encoding transcription factors) are known to be expressed during development and significantly downregulated in adulthood. This may explain why some genes are expressed at lower levels (e.g., *Atoh1* and *Jag2*) or no longer detected (*Foxj1*, *Scn11a*, and *Tmc2*) in adult hair cells.

Several previous studies used microarray and RNA-seq to examine the gene expression profiles of cochlear and vestibular hair cells from embryonic and neonatal mice ^9,11–14^ as well as hair cells in the inner ear and lateral lines of larval and adult zebrafish^11,23–27^. Comparison of our dataset with the transcriptome datasets from previous studies offers another way to validate our results. The gene names and their expression values from microarray-based transcriptomes of IHCs and OHCs from adult mice^9^ are presented in Data Citation 7. Although the expression values are not directly comparable because of the two different techniques used, the majority of the genes that are detected in hair cells in different datasets are highly consistent. In the same file, we also included transcriptome datasets from neonatal mouse hair cells^12,13^. Since these datasets were obtained from a mixed population of both IHCs and OHCs from neonatal mice, some differences between the datasets are expected.

**Figure 4:**
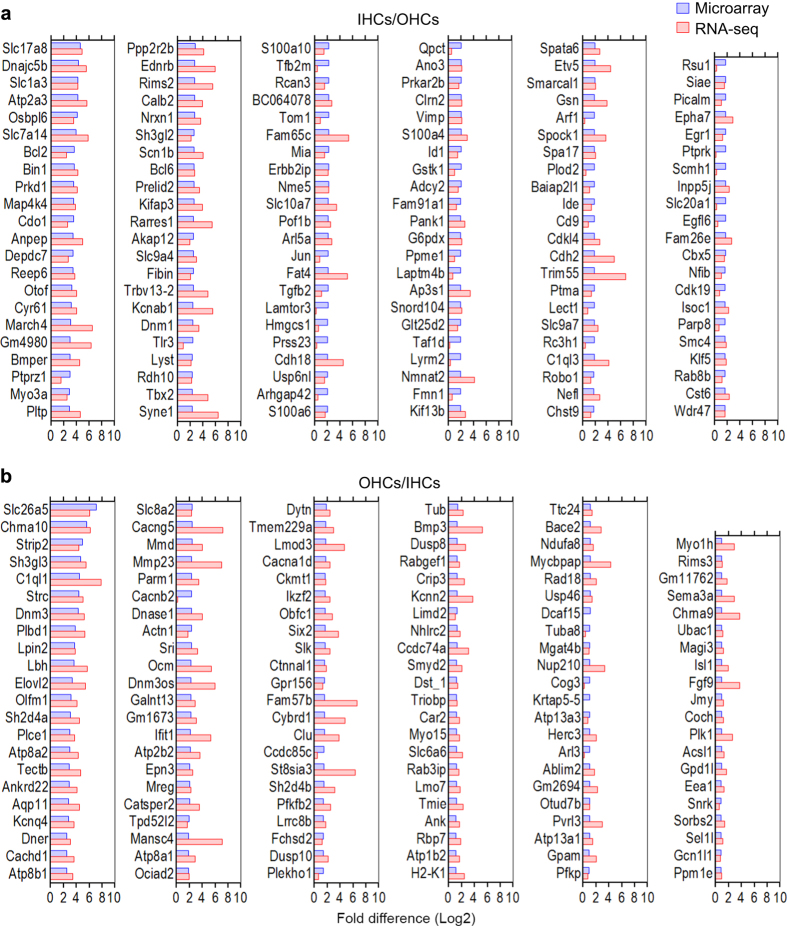
Comparison of differentially expressed genes in IHCs and OHCs quantified by microarray and RNA-seq techniques. The microarray data are based on genes presented in Fig. 6 of Liu *et al*.^9^ (**a**) Log2 fold difference between IHCs and OHCs (IHCs/OHCs). (**b**) Log2 fold difference between OHCs and IHCs (OHCs/IHCs).

Although the expression values from microarray and RNA-seq are not directly comparable, we expect that the genes that are differentially expressed in one cell population in the two studies should largely be consistent. We used the top differentially expressed genes in IHCs and OHCs from Fig. 4b,c of the microarray study^9^ for comparison. We computed the log2 fold difference between the two hair cell types from each study and present side-by-side comparison of the fold difference values from the two techniques in Fig. 4. As shown, none of the differentially expressed genes in IHCs (IHCs/OHCs in Fig. 4a) or OHCs (OHCs/IHCs in Fig. 4b) displace fold changes in the opposite direction from the two studies, suggesting that the differentially expressed genes identified by the two techniques are highly consistent. These differentially expressed genes may provide valuable information to understand different biological properties (such as structural and functional differences) of IHCs and OHCs in the adult inner ear.

## Usage notes

While acquired deafness associated with age or noise exposure is more common than genetic deafness by roughly two orders of magnitude, congenital deafness occurs in 1 out of every 1,000 to 2,000 births. Hereditary hearing loss and deafness can be regarded as syndromic or non-syndromic. Mutations or deficiencies affecting approximately 140 genes have been linked to inherited syndromic or non-syndromic hearing loss^16^. Although majority of these genes are known to be expressed in the inner ear, it is important to determine whether they are expressed in hair cells. We analyzed the expression of 125 known deafness genes. Table 4(available online only) shows expression levels of the 125 deafness genes in adult IHCs and OHCs. As shown, most of these genes are detected in hair cells. We should point out that several genes are known to be expressed during development and significantly downregulated in adulthood. Other genes may be expressed in spiral ganglion neurons, supporting cells, and stria vascularis and play important roles in those cells. Thus, it is not surprising that the expression of some genes is not detected in hair cells. However, the analysis will be highly useful for assisting the auditory research community in exploring the function of these deafness-related genes in hair cells.

## Additional information

**How to cite this article** Li, Y. *et al*. Transcriptomes of cochlear inner and outer hair cells from adult mice. *Sci. Data*. 5:180199 doi: 10.1038/sdata.2018.199 (2018).

**Publisher’s note** Springer Nature remains neutral with regard to jurisdictional claims in published maps and institutional affiliations.

## Supplementary Material



## Figures and Tables

**Table 1:** Sequences of oligonucleotide primers for q-PCR.

GENE	FOWARD PRIMER	REVERSE PRIMER
*Bcl2*	ACGTGGACCTCATGGAGTG	TGTGTATAGCAATCCCAGGCA
*Bcl6*	GTG TCC TGG GGT TAC AGG TG	CCT GTC CTG CCT ACC CAT AG
*Dnajc5b*	ATTTTTGTTGCTGCCTTTGC	AGGCTGGAGAACAACTGGAA
*Ednrb*	AAAGCCAACGATCACGGATA	CCTTTCTGCTAGCATGGTTTTT
*Fzd4*	TGCCAGAACCTCGGCTACA	ATGAGCGGCGTGAAAGTTGT
*Hdac3*	GCCAAGACCGTGGCGTATT	GTCCAGCTCCATAGTGGAAGT
*Otof*	GGC GCT TCA TIT ATC CTT TCG AC	GAC GAG GTG CCG GAT TGC CTT TAG C
*Prkd1*	GGGGGCATCTCGTTCCATC	GTGCCGAAAAAGCAGGATCTT
*Slc17a8*	GGAGACAGAACTCAACCACGA	TTCGGCCTGGTAGGATAATG
*Slc1a3*	GCACCAAGTGTTGGAAACTG	TTCAAATGTAGGCTAAAACCGATA
*Slc7a14*	CACCCTGGTCTCTGTCTGTG	CTGGAAAATTCCTCCCCTTC
*Tbx2*	CCGATGACTGCCGCTATAAGT	CCATCCACTGTTCCCCTGT
*Nono*	GCCAGAATGAAGGCTTGACTAT	TATCAGGGGGAAGATTGCCCA
*Ppia*	GAGCTGTTTGCAGACAAAGTTC	CCCTGGCACATGAATCCTGG
*C1ql1*	GGGGCAACAGCAACAAATAC	CCTTGGTCAGGCAATTTGAA
*Carhsp1*	CCCACGCATCAGACTTCTGTA	GTAGGCAGAGGGCTAGGGA
*Chrna9*	CGTGTGATCTCCACCAGTGT	TCCTTCATCCCTTTATCCTTGA
*Chrna10*	AGCCCTTCTGCATCACGTAG	AAAGCGGTCCATTACTCTGG
*Clu*	CCATCTGCAACTAGCTGTGAG	TCCGTTTTCTTCGGAAGTAAGAC
*Dmn3*	AACTTCACATCAACGCGACC	CTCGCACTGGAGTCTCTGAT
*Isl1*	ATGATGGTGGTTTACAGGCTAAC	TCGATGCTACTTCACTGCCAG
*Kcnq4*	GCTCAACCAAGTCCGCTTC	GTGATCGGAATCAGCCACAGT
*Lbh*	CTGCTCTGACTATCTGAGATCGG	CGGTCAAAGTCTGATGGGTCC
*Lmo7*	TTGAAACAACGGATTTTCGAGC	GACGCCAGGTTTGAGCTTATT
*Lmod3*	TTCAAGATGGGCAGCTAGAAAAT	TCGTCAGCTCGGAGATAGGAA
*Six2*	CACCTCCACAAGAATGAAAGCG	CTCCGCCTCGATGTAGTGC
*Slc26a5*	CACTCATTATGGGAGCGAGA	TCCGTCTACTTCTGCATCCAC
*Strip2*	ACTGGGGTCCTGAGTCAAAG	TTGACAAAAGTGAGCAGGGC

**Table 2:** Accession numbers for each biological sample.

Organism	Cell Type	Replicate	Analysis type	Sample ID	Raw data accession number
*Mus musculus*	1,000 IHCs	Biological Replicate 1, Technical replicate 1	RNA-Sequencing	IHC_1	SRX3757335
*Mus musculus*	1,000 IHCs	Biological Replicate 1, Technical replicate 2	RNA-Sequencing	IHC_2	SRX3757336
*Mus musculus*	1,000 IHCs	Biological Replicate 2, Technical replicate 1	RNA-Sequencing	IHC_3	SRX3757337
*Mus musculus*	1,000 IHCs	Biological Replicate 2, Technical replicate 2	RNA-Sequencing	IHC_4	SRX3757338
*Mus musculus*	1,000 OHCs	Biological Replicate 1, Technical replicate 1	RNA-Sequencing	OHC_1	SRX3757339
*Mus musculus*	1,000 OHCs	Biological Replicate 1, Technical replicate 2	RNA-Sequencing	OHC_2	SRX3757340
*Mus musculus*	1,000 OHCs	Biological Replicate 2, Technical replicate 1	RNA-Sequencing	OHC_3	SRX3757341
*Mus musculus*	1,000 OHCs	Biological Replicate 2, Technical replicate 2	RNA-Sequencing	OHC_4	SRX3757343
*Mus musculus*	1,000 OHCs	Biological Replicate 3, Technical replicate 1	RNA-Sequencing	OHC_5	SRX3757344
*Mus musculus*	1,000 OHCs	Biological Replicate 3, Technical replicate 2	RNA-Sequencing	OHC_6	SRX3757345

**Table 3:** Genes that have been verified by immunocytochemistry or *in situ* hybridization in previous studies.

**Gene**	**IHCs**	**OHCs**	**References**
*Anxa4*	11.37	9.91	14
*Atoh1*	0.63	0.66	28
*Atp7b*	0.03	0.01	29
*Barhl1*	3.77	4.55	30
*Bdnf*	17.1	8.37	31
*Cacnb2*	3.49	2.46	32
*Calb2*	5119.84	287.3	33
*Calm1*	2250.57	949.8	34
*Casz1*	0.54	0.11	12
*Cdh23*	3.51	6.83	35
*Celsr3*	0.44	0.77	36
*Chrm4*	0	0	37
*Chrna9*	28.34	325.34	38
*Chrna10*	10.86	481.35	39
*Cldn14*	65.44	89.86	40
*Cldn9*	2004.27	1694.26	41
*Ctbp2*	9.59	9.55	42
*Dll1*	0.33	0	43
*Dll3*	0.04	0	44
*Eps8l2*	59.77	95.33	12
*Foxj1*	0	0.01	45
*Fscn2*	222.32	246.68	46
*Gfi1*	53.41	88.58	47
*Gpr98*	0	0	48
*Grp*	0	3.43	13
*Grxcr1*	28.8	49.91	49
*Grxcr2*	200.98	284.33	50
*Hes6*	10.4	30.47	51
*Jag2*	1.76	4	52
*Kcnh6*	0.82	0.03	12
*Kcnq4*	3.61	40.14	53
*Kif21b*	0.01	0.01	12
*Lbh*	0.6	15.94	20
*Lhfpl5*	741.86	1019.74	54
*Lhx3*	9.58	20.79	55
*Lmo1*	91.38	31.07	56
*Lmod1*	6.77	10.1	13
*Lmod3*	3.13	63.55	13
*Loxhd1*	8.09	14.62	57
*Mcoln3*	130.95	179.07	58
*Mfng*	0	0.39	12
*Mgat5b*	2.61	9.01	12
*Mreg*	11.1	39.48	12
*Mycl1*	3.29	2.4	12
*Myo3a*	0.63	0.66	59
*Myo3b*	0.03	0.01	60
*Myo6*	3.77	4.55	61
*Myo7a*	17.1	8.37	62
*Naca*	3.49	2.46	12
*Nhlh1*	5119.84	287.3	63
*Otof*	2250.57	949.8	64
*Pacsin1*	0.54	0.11	12
*Pcdh15*	3.51	6.83	65
*Pcp4*	0.44	0.77	66
*Pou4f3*	0	0	67
*Ptgir*	10.86	481.35	12
*Ptprq*	28.34	325.34	68
*Pvalb*	65.44	89.86	69
*Pvrl1*	0.63	0.52	70
*Rab11fip1*	9.59	9.55	12
*Rab15*	0.33	0	71
*Rasd2*	0.04	0	14
*Rassf4*	59.77	95.33	71
*Rbm24*	0	0.01	12
*Rfx3*	222.32	246.68	11
*Scn11a*	53.41	88.58	12
*Selm*	130.44	70.94	71
*Sema5b*	0	3.43	12
*Slc17a8*	28.8	49.91	72
*Slc26a5*	200.98	284.33	73
*Slc6a11*	10.4	30.47	12
*Smpx*	1.76	4	74
*Srrm4*	0.82	0.03	12
*Sstr2*	3.61	40.14	75
*Stard10*	0.01	0.01	12
*Strc*	0.6	15.94	76
*Strip2*	741.86	1019.74	13
*Thsd7b*	9.58	20.79	12
*Tmc1*	91.38	31.07	77
*Tmc2*	6.77	10.1	77
*Tmie*	3.13	63.55	78
*Tomt*	8.09	14.62	79
*Ttc21a*	130.95	179.07	12
*Umodl1*	0	0.39	12
*Ush1g*	2.61	9.01	80
*Ush2a*	11.1	39.48	81

**Table 4:** The expression of genes that are known to cause hearing loss in humans.

**Feature ID**	**Locus**	**IHCs**	**OHCs**
*Actg1*	DFNA20/26	386.6	264.65
*Adcy1*	DFNB44	0.4	0.05
*Adgrv1*	Usher 2 C	0.47	0.44
*Aifm1*	DFNX5	44.95	45.86
*Bdp1*	DFNB49	2.33	2.87
*Bsnd*	DFNB73	0.04	0.23
*Cabp2*	DFNB93	1347.69	635.28
*Ccdc50*	DFNA44	3.39	2.5
*Cd164*	DFNA66	87.93	97.7
*Cdc14a*	DFNB32/105	10.54	26.47
*Cdh23*	DFNB12	3.51	6.83
*Ceacam16*	DFNA4B	1756.23	3291.72
*Chd7*	CHARGE Synd.	3.99	8.54
*Cib2*	DFNB48	1102.26	972.84
*Cldn14*	DFNB29	65.44	89.86
*Clic5*	DFNB103	9.61	20.08
*Clpp*	DFNB81	77.79	59.14
*Clrn1*	Usher 3 A	72.78	84.28
*Coch*	DFNA9	46.91	105.2
*Col11a1*	Stickler Synd. type II	0.69	0.25
*Col11a2*	DFNB53	2.64	3.3
*Col2a1*	Stickler Synd.	0.28	0.68
*Col4a3*	Alport Synd.	0	0.01
*Col4a4*	Alport Synd.	0.11	0.05
*Col4a5*	X-linked Alport Synd.	0.84	1.52
*Col4a6*	DFNX6	0.01	0.07
*Col9a1*	Stickler Synd.	1.2	1.91
*Col9a2*	Stickler Synd.	4.15	6.87
*Crym*	DFNA40	0.12	0.15
*Dcdc2b*	DFNB66	1.28	0.48
*Dfna5*	DFNA5	0.62	0.27
*Dfnb59*	DFNB59	162.27	101.56
*Diablo*	DFNA64	20.62	21.74
*Diap1*	DFNA1	2.71	2.21
*Diap3*	AUNA1	0.56	0.08
*Dmxl2*	DFNA71	3.65	1.83
*Dspp*	DFNA39	0	0
*Edn3*	WS4B	0	0.02
*Ednrb*	WS4A	34.28	0.55
*Elmod3*	DFNB88	18.99	13.3
*Eps8*	DFNB102	6.09	9.91
*Eps8l2*	DFNB106	59.77	95.33
*Eral1*		13.91	14.2
*Espn*	DFNB36	38.37	48.63
*Esrp1*		3.82	3.11
*Esrrb*	DFNB35	0	0
*Eya1*		13.26	10.44
*Eya4*	DFNA10	7.24	12.32
*Fam65b*	DFNB104	5.47	4.61
*Foxi1*		0	0
*Gipc3*	DFNB95	11.8	9.09
*Gjb2*	DFNB1A	405.43	120.57
*Gjb3*	DFNA2B	0	0
*Gjb6*	DFNB1B	446.52	393.09
*Gpsm2*	DFNB82	83.17	56.65
*Grhl2*	DFNA28	2.62	2.09
*Grxcr1*	DFNB25	28.8	49.91
*Grxcr2*	DFNB101	200.98	284.33
*Hars2*	Perrault Synd.	14.31	11.57
*Hgf*	DFNB39	0	0.05
*Homer2*	DFNA68	26.7	42.9
*Hsd17b4*	Perrault Synd.	19.03	15.7
*Ildr1*	DFNB42	31.14	58.72
*Kars*	DFNB89	56.19	36.7
*Kcne1*	Jervell and Lange-Nielsen Synd.	0.04	1.01
*Kcnj10*		18.78	28.56
*Kcnq1*	Jervell and Lange-Nielsen Synd.	0.96	0.76
*Kcnq4*	DFNA2A	3.61	40.14
*Kitl*	WS2	4.52	0.31
*Lars2*		19.45	13.16
*Lhfpl5*	DFNB66/67	741.86	1019.7
*Loxhd1*	DFNB77	8.09	14.62
*Marveld2*	DFNB49	34.59	21.2
*Mcm2*	DFNA70	1.15	1.14
*Met*	DFNB97	0.4	0.52
*mir96*	DFNA50	0	0
*Mitf*	WS2	0.18	0.3
*Msrb3*	DFNB74	63.23	81.72
*Mt-rnr1*		402.58	498.24
*Mt-te*		4.38	2.95
*Mt-tk*		0	0
*Mt-tl1*		0.3	0.4
*Mt-ts1*		0	0.24
*Myh14*	DFNA4	2.76	2.69
*Myh9*	DFNA17	0.46	0.47
*Myo1a*	DFNA48	0.03	0.05
*Myo3a*	DFNB30	9.2	1.51
*Myo6*	DFNB37	103	100.7
*Myo7a*	DFNB2	22.01	15.47
*Myo15*	DFNB3	9.13	28.27
*Nars2*	DFNB94	5.94	6.3
*Ndp*	Norrie Disease	1.64	0.55
*Nlrp3*	DFNA34	0	0
*Osbpl2*	DFNA67	21.77	21.28
*Otoa*	DFNB22	8.37	3.58
*Otof*	DFNB9	210.17	13.79
*Otog*	DFNB18B	19.07	45.92
*Otogl*	DFNB84	4.79	7.24
*P2rx2*	DFNA41	102.08	289.54
*Pax3*	WS1,2	0	0
*Pcdh15*	DFNB23	0.73	1.4
*Pdzd7*		1	6.27
*Pnpt1*	DFNB70	3.44	6.35
*Pou3f4*	DFNX2 (DFN3)	0.42	0.52
*Pou4f3*	DFNA15	110.96	86.69
*Prps1*	DFNX1 (DFN2)	12.58	12.39
*Ptprq*	DFNB84	18.51	7.51
*Rdx*	DFNB24	26.91	33.85
*Ror1*		0.38	0.03
*S1pr2*	DFNB68	12.06	17.22
*Sema3e*	CHARGE Synd.	0.05	0.2
*Serpinb6a*	DFNB91	38.55	36.37
*Six1*	DFNA23	30.95	39.19
*Six5*		2.06	1.76
*Slc17a8*	DFNA25	50.17	2.81
*Slc22a4*	DFNB60	0	0.13
*Slc26a4*	DFNB4	2.04	10.9
*Slc26a5*	DFNB61	9.51	548.95
*Slitrk6*		43.09	33.92
*Smpx*	DFNX4 (DFN6)	253.76	231.97
*Snai2*		0	0.3
*Sox10*	WS2	26.68	13.4
*Strc*	DFNB16	1.79	42.47
*Syne4*	DFNB76	151.04	280.92
*Tbc1d24*	DFNB86	0.11	0.52
*Tcof1*	Treacher-Collins-Franceschetti Synd.	2.45	7.88
*Tecta*	DFNB21	0.63	0
*Tjp2*	DFNA51	14.16	22.41
*Tmc1*	DFNB7/11	72.94	138.09
*Tmem132e*	DFNB99	0	0
*Tmie*	DFNB6	11.46	46.5
*Tmprss3*	DFNB8/ 10	499.87	417.15
*Tnc*	DFNA56	0	0.26
*Tomt*	DFNB63	76.5	54.46
*Tprn*	DFNB79	2.16	3.05
*Triobp*	DFNB28	26.11	59.16
*Tspear*	DFNB98	0.13	0.05
*Ush1c*	DFNB18	106.76	93.33
*Ush1g*	USH1G	2.85	5.53
*Ush2a*	USH2A	0	0.01
*Wbp2*		212.73	143.01
*Wfs1*	DFNA6/14/38	9.4	9.39
*Whrn*	DFNB31	18.89	0.98
